# Combined adenocarcinoid and mucinous cystadenoma of the appendix: a case report

**DOI:** 10.1186/1752-1947-3-28

**Published:** 2009-01-26

**Authors:** A Velusamy, S Saw, J Gossage, STR Bailey, J Schofield

**Affiliations:** 1 Department of Surgery, Maidstone Hospital, Maidstone ME16 9QQ, UK; 2 Department of Pathology, Preston Hall Hospital, Aylesford, Kent ME20 7NJ, UK

## Abstract

**Introduction:**

Adenocarcinoid of the appendix is a rare malignant tumour with features of both adenocarcinoma and carcinoid, showing both epithelial and endocrine differentiation. Mucinous cystadenoma is the commonest of the benign neoplasms of the appendix, with an incidence of 0.6% in appendicectomy specimens. We report a rare combination of these tumours and discuss the latest treatment options. To the best of our knowledge, only six cases have been reported in the literature to date.

**Case presentation:**

A 71-year-old Caucasian man presented to our department with a right iliac fossa mass associated with pain. Laparoscopy revealed an adenocarcinoid of the appendix in combination with mucinous cystadenoma. He underwent a radical right hemicolectomy with clear margins and lymph nodes.

**Conclusion:**

Adenocarcinoids account for 2% of primary appendiceal malignancies. Most tumours are less than 2 cm in diameter and 20% of them metastasize to the ovaries. The mean age for presentation is 59 years and the 5-year survival rate ranges from 60% to 84%. Right hemicolectomy is generally advised if any of the following features are present: tumours greater than 2 cm, involvement of resection margins, greater than 2 mitoses/10 high-power fields on histology, extension of tumour beyond serosa. Chemotherapy mostly with 5-Fluorouracil and Leucovorin is advised for remnant disease after surgery. Cytoreductive surgery with intraperitoneal chemotherapy can offer improved survival for advanced peritoneal dissemination.

## Introduction

Mucinous cystadenoma is the commonest of the benign neoplasms of the appendix, with an incidence of 0.6% in appendicectomy specimens [1]. Adenocarcinoid is a rare but well recognised tumour of the appendix which exhibits features of both carcinoid and adenocarcinoma. We report a patient with combined adenocarcinoid and mucinous cystadenoma of the appendix. To the best of our knowledge, only six cases have been reported in the literature to date [2,3].

## Case presentation

### Case report

**Figure 1:**
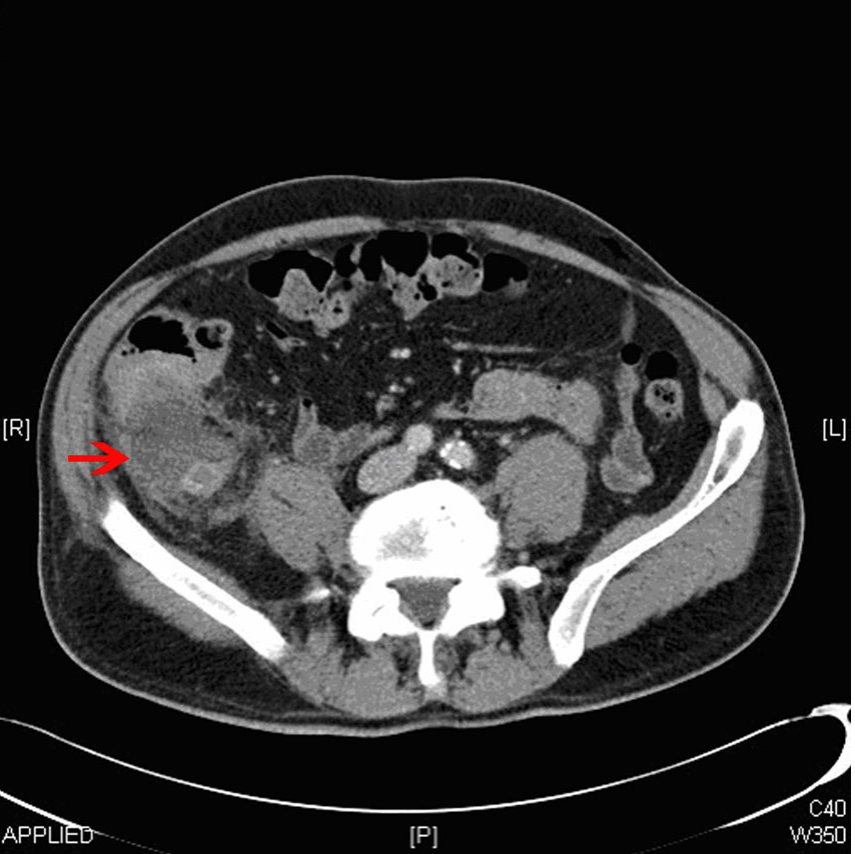
**Computed tomography scan showing appendix mucocele**.

A 71-year-old man presented to the surgical department with a 2-week history of colicky right iliac fossa pain and a 1-week history of diarrhoea. He did not have rectal bleeding or weight loss. On examination, he was found to have a right iliac fossa mass. Serum carcinoembryonic antigen (CEA) was <2 μg/L and the computed tomography (CT) scan revealed an appendiceal mass (Figure 1) without any surrounding lymph node enlargement. Laparoscopy showed an appendiceal mass with mucinous discharge and a laparoscopic appendicectomy was performed. Histology revealed both an adenocarcinoid involving the resected margin and a mucinous cystadenoma of the tip. The patient subsequently underwent a laparoscopic radical right hemicolectomy. The right colon and all of the 15 lymph nodes resected were clear of tumour.

### Pathology

**Figure 2:**
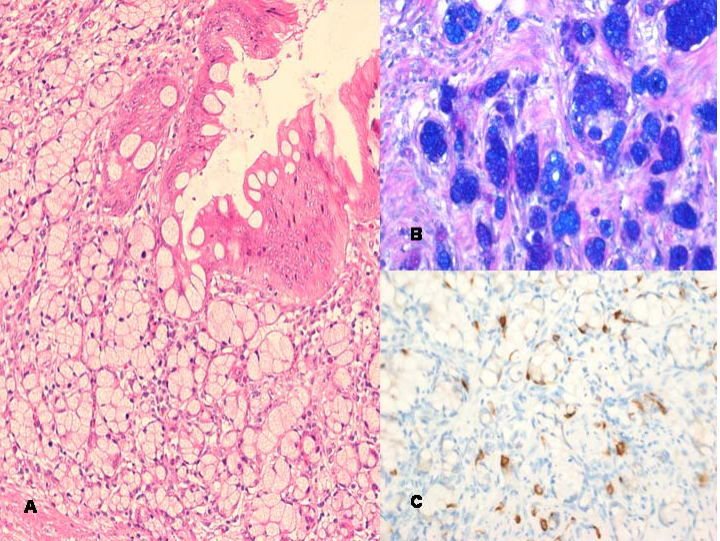
**(A) Mucinous cystadenoma and goblet cell carcinoid with hematoxylin and eosin (×200)**. (B) Goblet cell mucin positive with alcian blue/diastase/periodic acid Schiff stain (×200). (C) Chromogranin immunohistochemistry positive (×200).

Macroscopically, the normal anatomy of the appendix was distorted with dilatation and mucocele formation. Histology revealed a mucinous cystadenoma involving the tip of the appendix. Proximally, the appendix was infiltrated by adenocarcinoid composed of islands of epithelial cells with abundant intracellular mucin and eosinophilic granules (Figure 2). The tumour measured at least 12 mm in maximum diameter with extension to the serosal surface and into the mesoappendix. The tumour was present at the surgical resection margin. Mucin stains were positive and immunohistochemical stains showed strong positivity for chromogranin, CD56 and CK20, whilst CK7 was negative. The resected right colon showed no residual tumour and the lymph nodes were not involved.

## Conclusion

Adenocarcinoid is recognised as a tumour which has features of both adenocarcinoma and carcinoid, first described by Gagne *et al. *in 1969 [4]. It is widely known by different names such as goblet cell carcinoid [5], crypt cell carcinoid, and amphicrine cell carcinoma. There is still debate about the origin of this tumour and several hypotheses exist, hence the various nomenclature. Adenocarcinoma arises from primordial endodermal elements while carcinoid arises from neural crest cells. Some authors suggest that goblet cell carcinoids arise from a pluripotent cell with divergent neuroendocrine and mucinous differentiation [6].

Mucinous cystadenoma is rare but is the commonest of the benign appendiceal tumours. It can present as appendicitis, mucocele or if the tumour ruptures, as pseudomyxoma peritonei. Treatment is usually appendicectomy with care taken not to spill the mucin intraperitoneally.

Adenocarcinoids account for 2% of primary appendiceal malignancies. Most tumours are less than 2 cm in diameter and 20% of them metastasize to the ovaries [7]. The mean age at presentation is 59 years and the 5-year survival rate ranges from 60% to 84% [8]. The tumour usually spares the mucosa, infiltrates muscularis propria and peri-appendiceal fat and can stain positively for mucin, CEA, cytokeratin, lysozyme, chromogranin A, serotonin and synaptophysin. Goblet cell carcinoids have increased expression of NAP1L1, MAGE-D2, and MTA-1 genes compared with benign carcinoids. Ki 67 is a tumour marker which is expressed in higher levels in metastatic adenocarcinoids compared to localized ones. This can be useful in predicting tumour behaviour and subsequent surgical management [9]. Recent studies suggest that goblet cell carcinoids have biological and immunohistochemical profiles more similar to adenocarcinoma than to classical carcinoids which may explain their more aggressive behaviour and therefore substantiate more extensive treatment [10,11].

There is ongoing surgical controversy as to whether appendicectomy or right hemicolectomy is necessary for appendiceal carcinoid tumours. For classical carcinoid tumours, most centres consider tumour size to be the main discretionary factor. Those <2 cm are usually treated with appendicectomy alone. For adenocarcinoids, right hemicolectomy is generally advised if any of the following features are present: tumours greater than 2 cm, involvement of resected margins, greater than 2 mitoses/10 high-power fields (hpf), extension of tumour beyond serosa, lymphovascular invasion or lymph node metastases [12,13]. Some authors recommend right hemicolectomy for adenocarcinoids of any size due to their propensity to metastasize [7], while others use similar determinative histological criteria as those for classical carcinoids to plan treatment [13]. A meta-analysis of retrospective chart reviews by Varisco *et al. *evaluated the efficacy of appendicectomy versus hemicolectomy for localized adenocarcinoids. Their analysis suggests appendicectomy alone has a role in the treatment of localized tumours. However, in the presence of unfavourable features including moderate to severe atypia, involvement of caecum, and more than 2 mitoses per hpf, they recommend an interval hemicolectomy [14].

Chemotherapy mostly with 5-Fluorouracil and Leucovorin is advised for remnant disease after surgery. Cytoreductive surgery with intraperitoneal chemotherapy can offer improved survival for advanced peritoneal dissemination [8]. In female patients regardless of age, bilateral salpingo-oopherectomy is advocated, as there is a significant risk of ovarian involvement. Irrespective of the type of surgical intervention, all patients warrant lifelong colonoscopic surveillance.

## Consent

Written informed consent was obtained from the patient for publication of this case report and any accompanying images. A copy of the written consent is available for review by the Editor-in-Chief of this journal.

## Competing interests

The authors declare that they have no competing interests.

## Authors' contributions

AV contributed in the conceptualization and design of the manuscript, literature search, data acquisition and manuscript preparation. SS was involved in data acquisition, illustration and manuscript preparation. JG contributed in the design of the manuscript, data analysis, editing and manuscript review. JS was involved in the literature search, data acquisition and revising the manuscript critically for content. SB contributed to the drafting of the manuscript, literature review, editing and was the clinician responsible for making the treatment decisions for the patient. All authors read and approved the final manuscript.

## References

[B1] Marudhanayagam R Williams GT Rees BI Review of the pathological results of 2660 appendicectomy specimens J Gastroenterol 2006 41 8 745 749 10.1007/s00535-006-1855-5 16988762

[B2] Al-Talib RK Mason CH Theaker CJ Combined goblet cell carcinoid and mucinous cystadenoma of the appendix J Clin Pathol 1995 48 869 870 10.1136/jcp.48.9.869 7490325 PMC502880

[B3] Carr NJ Remotti H Sobin LH Dual carcinoid/epithelial neoplasia of the appendix Histopathology 1995 27 6 557 562 10.1111/j.1365-2559.1995.tb00327.x 8838336

[B4] Gagne F Fortin P Dufour V Delage C Tumeurs de l'appendice associant de carreteres histologique de carcinoide et d'adenocarcinome Ann Anat Pathol 1969 14 393 406 5378353

[B5] Subbuswamy SG Gibb NM Ross CF Morson BC Goblet cell carcinoid of the appendix Cancer 1974 34 338 344 10.1002/1097-0142(197408)34:2<338::AID-CNCR2820340218>3.0.CO;2-W 4852178

[B6] Kanthan R Saxena A Kanthan SC Goblet cell carcinoids of the appendix: immunophenotype and ultrastructural study Arch Path Lab Med 2001 125 386 390 11231488 10.5858/2001-125-0386-GCCOTA

[B7] Staley CA Morris PJ, Wood WC Primary appendiceal malignancies Oxford Textbook of Surgery 2000 section 27.2 2 New York: Oxford University Press 1545 1548

[B8] Pahalavan PS Kanthan R Goblet cell carcinoid of the appendix World J Surg Oncol 2005 3 366 10.1186/1477-7819-3-36 PMC1182398 15967038

[B9] Modlin IM Kidd M Latich I Zikusoka MN Eick GN Mane SM Camp RL Genetic differentiation of appendiceal tumor malignancy: a guide for the perplexed Ann Surg 2006 244 1 52 60 10.1097/01.sla.0000217617.06782.d5 16794389 PMC1570599

[B10] van Eeden S Offerhaus GJ Hart AA Boerrigter L Nederlof P Porter E van Velthuysen M-LF Goblet cell carcinoid of the appendix: a specific type of carcinoma Histopathology 2007 51 6 763 773 10.1111/j.1365-2559.2007.02883.x 18042066

[B11] Alsaad KO Serra S Schmitt A Perren A Chetty R Cytokeratins 7 and 20 immunoexpression profile in goblet cell and classical carcinoids of appendix Endocr Pathol 2007 18 1 16 22 10.1007/s12022-007-0004-x 17652796

[B12] Fornaro R Frascio M Sticchi C De Salvo L Stabilini C Mandolfino F Ricci B Gianetta E Appendectomy or right hemicolectomy in the treatment of appendiceal carcinoid tumours? Tumori 2007 93 6 587 590 18338494 10.1177/030089160709300612

[B13] Bucher P Gervaz P Ris F Oulhaci W Egger JF Moerel P Surgical treatment of appendiceal adenocarcinoid World J Surg 2005 29 11 1436 1439 10.1007/s00268-005-7958-y 16136284

[B14] Varisco B McAlvin B Dias J Franga D Adenocarcinoid of the appendix: is right hemicolectomy necessary? A meta-analysis of retrospective chart reviews Am Surg 2004 70 7 593 599 15279181

